# Brazil: rapid progress and the challenge of inequality

**DOI:** 10.1186/s12939-016-0465-y

**Published:** 2016-11-17

**Authors:** Michael Marmot

**Affiliations:** UCL Institute of Health Equity, Department of Epidemiology and Public Health, London, UK

If one accepts the argument that health is a good measure of how a country is doing socially, then Brazil has come a huge distance. In the 1950s, male life expectancy in Brazil was about 25 years shorter than in the US. In 2014, it was about 6 years shorter. UNDP [1] Human Development Reports are helpful in showing quite how far Brazil has travelled along a path of development. Continuing with life expectancy as a metric of social progress, currently the range, for both sexes, is from 49 in Swaziland to 83.5 in Japan, now pipped by 84 in Hong Kong. On that scale, Brazil at 74.5 is a good deal closer to Japan than it is to Sub-Saharan Africa – 9 years behind Japan, 25 years ahead of the worst in Africa.

There are other ways we can see quite how truly impressive has been Brazil’s social progress, yet how far it still has to go. It is instructive to compare Brazil not with sub-Saharan Africa but with a South American country that lags behind it, Paraguay, for example. Maternal mortality should be more or less completely avoidable. In Norway it is 4 per 100,000; in Brazil 69; in Paraguay 110. Reverting to Africa, In Nigeria it is 560. Again, Brazil is a good deal closer to the best than to the worst.

Turning to social conditions at work, one marker of an advanced economy is that childhood means childhood. Children do not need to carry the burden of household chores or work for economic gain outside the home. UNDP defines child labour as percentage of children aged 5–11 who did at least one hour of economic activity in a week or at least 28 h of household chores, or children aged 12–14 who did at least 14 h of economic activity or at least 28 h of household chores. In Norway the percent of children engaged in child labour is 0; in Brazil it is 8.3 %; in Paraguay 27.6 %.

Work, of course, should be a way out of poverty. In rich countries, no one in work is on $2 a day, adjusting for purchasing power. In Brazil, 3 % are; in Paraguay 8 %.

And when work is finished? In Norway, 100 % of people of pensionable age receive a pension; in Brazil 86 %; in Paraguay 22 %.

All of these figures support the thesis that Brazil has made great strides towards the advanced level of social conditions and programmes that are responsible for the good health enjoyed by many European countries. But one striking characteristic holds Brazil back: the benefits of social progress are not enjoyed equally. One measure of economic inequality is the quintile ratio: the ratio of the average income of the richest 20 % of the population to the average income of the poorest 20 % of the population. The benchmark is Norway, where the ratio is 4.0; in the US it is 9.8; in Brazil it is 16.9 which is even higher than the figure in Paraguay, 13.

While I am not suggesting an inevitable relation between income inequality and overall health – after all Brazil has better health than Paraguay, despite Brazil having greater inequality – inequality matters. Homicide rates have been linked to inequality within Brazil, and the homicide rates in Brazil are high: 25/100,000 in Brazil, compared to 4.7 in the US, and less than 1 in Switzerland.

More generally health tracks social and economic inequalities. The papers in this issue illustrate. They show clearly that non-communicable diseases are more common in people of lower social position. Given the implementation of the Brazilian Unified Health System there is understandable interest in the degree to which it has eliminated inequalities in access – slightly mixed picture.

In the future we should look to more analyses on trends in the social determinants of health. Brazil, of course, is interesting here. It had a Brazilian Commission on Social Determinants of Health. I am careful not to jump to conclusions of cause and effect, but it is of great interest that the gini coefficient of income inequality has diminished. One contribution will have been from Bolsa Familia the Brazilian conditional cash transfer programme. It has covered a quarter of the Brazilian population and is credited with reducing the estimated poverty rate in Brazil by 8 percentage points. There has also been an increase in school enrolment. These are remarkable achievements. We will watch with great interest if Bolsa Familia and other social programmes not only contribute to Brazil’s continued health improvement but also reduce inequalities.

Of central importance will be development of systems to monitor health inequalities and social determinants. The next generation of papers such as these will, one hopes, be able to draw on such improved possibilities for measurement. Brazil has shown the way in other important respects. We look for it to do so here, too.
